# Improving the Quality of Junior Doctor Handover in Tyrone and Fermanagh Hospital, Northern Ireland

**DOI:** 10.1192/bjo.2024.367

**Published:** 2024-08-01

**Authors:** Jill Fulton, Archana C. Sreekumar, Aisling Sheridan

**Affiliations:** WHSCT Tyrone and Fermanagh Hospital, Omagh, United Kingdom

## Abstract

**Aims:**

To improve the quality of junior doctor handover in the Tyrone and Fermanagh hospital. The hospital is spread across a number of inpatient sites making it difficult to complete an in-person handover. Each day the handover is completed on a Word document and sent via trust email to relevant staff. Issues were identified with the quality of information shared and how the outstanding tasks were handed over.

**Methods:**

A PDSA cycle was implemented to explore outstanding issues with the handover and consider how change might be implemented. Junior doctors identified various issues including the lack of a common format, the amounts and relevancy of information shared and identifying an individual or team to conduct the outstanding tasks.

A baseline audit for a 3 month period (July–September 2023) was completed. Results were reviewed and a driver diagram was established. Suggestions identified for improvement included the use of new template and an in-person handover.

A new template for recording information was drawn up and agreed by the group. It included basic demographic prompts such as staff member on shift and the date of handover. The template included prompts for key patient information identified from initial audit as frequently forgotten.

The template was emailed to doctors on the rota and was also highlighted to new staff at junior doctor changeover points. This new template was the intervention chosen for re-audit between November 2023 and January 2024.

**Results:**

Following the application of our intervention, completion of the handover improved. From an information governance perspective the identification of staff and shift dates improved (to 98% & 99% respectively). The security of information shared improved through use of password (69% to 91%).

The quality of information sharing also improved with the percentage improvement of key demographics increasing, such as patient initials (29.4%), Healthcare number (9.2%), MHO status (15.46%), patient summary (19.76%) and working diagnosis (34.91%) and finally an increase of 88.74% in identifying the person for following up outstanding tasks.

**Conclusion:**

The use of a handover template has improved the quality of information shared across a number of key areas. The identification of person for handover has improved significantly with this tool and is felt to represent an improvement in patient safety. Following re-audit cycle, other areas were identified for further changes such as adjusting prompts on the template and a secure folder for storing the handover. These changes could be easily implemented in a subsequent audit cycle.

